# Estimating the global macroeconomic impact of colorectal cancer: evidence from Global Burden of Disease 2021 and Value of a Statistical Life Year framework

**DOI:** 10.1097/JS9.0000000000004687

**Published:** 2026-01-15

**Authors:** Wenwen Lv, Shihan Wang, Lei Duan, Yejun Wu, Xia Tian, Zhongxun Dong, Shuhua Xu, Bingshun Wang

**Affiliations:** a Institute of Clinical Medicine, RuiJin Hospital Lu Wan Branch, Shanghai Jiaotong University School of Medicine, Shanghai, China; b State Key Laboratory of Genetics and Development of Complex Phenotypes, Center for Evolutionary Biology, School of Life Sciences, Fudan University, Shanghai, China; c National Institute of Parasitic Diseases, Chinese Center for Disease Control and Prevention (Chinese Center for Tropical Diseases Research), Shanghai, China; d Public Health Department, Putuo district People’s Hospital, Shanghai, China

**Keywords:** colorectal cancer, DALYs, economic burden, GBD 2021, global health, VSLY

## Abstract

**Background:**

Colorectal cancer (CRC) is the third most commonly diagnosed cancer and the second leading cause of cancer-related death globally. However, its macroeconomic burden remains underexplored.

**Methods:**

Using Global Burden of Disease (GBD) 2021 data, we quantified the economic welfare loss due to CRC across 204 countries using the Value of a Statistical Life Year (VSLY) approach. The Valuation of a Statistical Life (VSL) was derived by adjusting the U.S. benchmark ($11.8 million) based on per capita gross domestic product (GDP) and income elasticity (base case: 1.0). VSLY was calculated by dividing VSL by half the national life expectancy. Country-specific Value of Lost Welfare (VLW) was estimated and expressed as a percentage of GDP (VLW/GDP). Aggregated analyses were performed by sociodemographic index (SDI) and GBD regions. Sensitivity analyses used alternative elasticity values (0.55, 1.5) and applied a 3% discount rate.

**Findings:**

In 2021, global CRC-related VLW was estimated at $3.49 trillion (95% uncertainty interval [UI]: $3.02–$3.96 trillion), equivalent to 2.28% (95% UI: 1.97%–2.58%) of global GDP. VLW/GDP ratios were highest in high-SDI regions (2.81%) and the Central Europe, Eastern Europe, and Central Asia super-region (3.48%). At the national level, VLW ranged from <$500 million in small island states to >$0.6 trillion in China and the U.S. Discounting increased VLW estimates by 35%–67%.

**Conclusions:**

CRC imposes a substantial and inequitable economic burden, particularly in economically developed and aging societies. Incorporating VSLY into cancer burden assessments underscores the urgency of investing in prevention, early detection, and surgical capacity strengthening, especially in middle-income and resource-limited settings.

## Graphical Abstract

**Figure:**
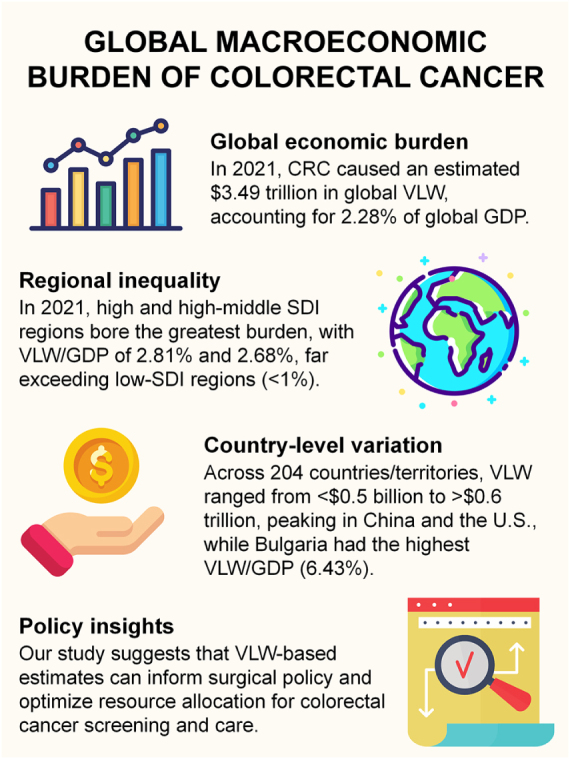

## Introduction

Colorectal cancer (CRC), the most common gastrointestinal malignancy, represents a significant and growing global public health challenge^[1]^. According to GLOBOCAN 2022, approximately 1.94 million new CRC cases and 895 000 related deaths occurred worldwide, ranking CRC third in cancer incidence and second in mortality^[2]^. CRC is typically characterized by an insidious onset, slow progression, and poor prognosis when diagnosed at advanced stages. Although surgical resection remains the cornerstone of curative treatment, many patients in low-resource settings present at unresectable or metastatic stages, limiting the potential benefits of surgery. Its burden extends beyond clinical outcomes, as reflected in the substantial and rising number of disability-adjusted life years (DALYs), particularly in countries undergoing demographic and epidemiological transitions^[3]^. Despite its increasing toll, current global burden estimates primarily focus on health outcomes, while the broader economic consequences remain underexplored^[4]^.

**HIGHLIGHTS**

- Global welfare losses due to colorectal cancer reached $3.49 trillion in 2021;
- High-sociodemographic-index regions had greater economic loss ratios despite better clinical outcomes;
- Among 204 countries, 102 have a discounted Value of Lost Welfare (VLW)-to-GDP ratio exceeding 2%;
- Central and Eastern Europe showed the highest VLW/GDP burden among all regions;
- Countries with aging populations experienced the greatest increases in CRC burden.

CRC arises from a multifactorial etiology involving complex interactions among genetic predisposition, environmental exposures, and behavioral risk factors^[5]^. Westernized dietary patterns, obesity, alcohol consumption, smoking, and chronic inflammatory bowel disease are strongly associated with increased CRC risk. Additionally, social determinants of health, including educational attainment, income level, and access to health services, critically influence risk exposure, timely diagnosis, and treatment outcomes^[6,7]^. Although recent advances in genomics and artificial intelligence have improved precision screening and therapeutic strategies, significant disparities in CRC outcomes persist across countries. Low- and middle-income countries (LMICs) continue to experience delayed diagnosis, inadequate screening coverage, and limited access to high-quality surgical and perioperative care^[8]^.

The economic burden of CRC is multifaceted. At the individual level, patients face considerable out-of-pocket costs from prolonged treatment, surgery, and surveillance. Indirect costs, including productivity loss and caregiving, often result in catastrophic expenditures, especially where insurance coverage is limited^[9,10]^. Nationally, CRC disproportionately affects the working-age population, contributing to labor force attrition and straining healthcare and welfare budgets. These consequences are further exacerbated by mental health challenges and intergenerational caregiving disruptions, yet they are often overlooked in national health accounts and macroeconomic planning^[7,11]^.

Most existing economic evaluations of CRC are limited in scope, focusing predominantly on direct medical costs. Few studies have adopted comprehensive macroeconomic approaches that capture productivity losses, human capital depreciation, and broader welfare impacts. Notably, Wang *et al* utilized a health-augmented macroeconomic model to estimate the global GDP loss attributable to 29 major cancers, including CRC, over the period 2020–2050. Their findings suggested that CRC alone may account for a cumulative GDP loss of $2.76 trillion, ranking second only to lung cancer in economic burden^[12]^. However, their analysis lacked cross-country detail needed for policy and resource planning. In recent years, the Value of a Statistical Life Year (VSLY) framework has gained traction in health economics, particularly in macroeconomic burden assessments for diseases such as stroke, cancer, and cardiovascular conditions^[13–17]^. Its application in oncology has also expanded, with studies using VSLY to quantify the economic value of survival gains and welfare losses associated with cancer mortality and morbidity^[14,18]^. By monetizing DALY losses, the VSLY approach enables more policy-relevant estimations of welfare loss across diverse socioeconomic settings.

In this study, we integrate DALY estimates from the Global Burden of Disease (GBD) 2021 study with macroeconomic indicators from the World Bank to quantify the monetary value of welfare losses attributable to CRC in 204 countries and territories. To our knowledge, this is the first study to apply the VSLY framework specifically to CRC at a global scale. Our analysis generates a cancer-specific economic profile that complements aggregate projections such as those by Wang *et al*^[12]^, while providing more granular insights into geographic variation in CRC’s macroeconomic impact. These findings support evidence-based prioritization and resource allocation, particularly in low-resource settings, and highlight the need to integrate economic evaluation into global cancer control strategies.

## Methods

### Data sources

This study integrated data from multiple authoritative sources. Estimates of CRC-related disease burden were obtained from the Global Burden of Disease Study 2021 (GBD 2021), led by the Institute for Health Metrics and Evaluation, providing country-, sex-, and age-specific DALYs for 204 countries and territories^[19,20]^. Macroeconomic indicators, including gross domestic product (GDP) per capita (purchasing power parity, PPP, constant 2021 Int$), were retrieved from the World Bank’s World Development Indicators^[12,13,16,17,21,22]^. The benchmark Value of a Statistical Life (VSL) was based on the U.S. Department of Transportation’s 2021 estimate of USD 11.8 million^[22,23]^. All data points were standardized to the year 2021 and expressed in international dollars (Int$) to enable cross-country comparability^[24]^.

### Economic valuation framework

Economic losses attributable to CRC were estimated using the Value of Lost Welfare (VLW) framework, using the VSLY as a monetary proxy for each DALY incurred. Country-specific VSLs were calculated using a proportional scaling method based on the United States benchmark, applying the formula:

$$VSL_{i}=VSL_{USA}\times {\left(\frac{GDP_{i}}{GDP_{USA}}\right)}^{\in }$$

where $VSL_{USA}$ is set at $11.8 million (2021 USD), $GDP_{i}$ is the per capita GDP (PPP, constant 2021 Int$) for country i, and ε denotes the income elasticity (IE) of VSL^[12,13,21]^. For the main analysis, ε was set to 1.0, with sensitivity analyses conducted using values of 0.55 and 1.5 to reflect different assumptions about willingness-to-pay across income levels. Each country’s VSLY was estimated by dividing the VSL by half of the national life expectancy, approximating the average remaining life years^[23]^.

### Calculation of country-level and relative economic burden

For each country *i*, the total welfare loss (Loss_i_) was estimated using the following formula:

$${\text{Loss}}_{\text{i}}{\text{=DALY}}_{\text{i}}\times \text{VSLY}$$

where $DALY_{i}$represents the total number of disability-adjusted life years due to CRC in country i, and $VSLY_{i}$ denotes the country-specific value of a statistical life year. All monetary estimates were converted to constant 2021 international dollars (Int$) using purchasing power parity (PPP) adjustments to ensure cross-country comparability.

To assess the relative economic impact, we further estimated the proportion of GDP lost:

$$\%{\text{GDP}}_{\text{loss},\text{i}}=({\text{Loss}}_{\text{i}}/{\text{GDP}}_{\text{i}})\times 100$$

This relative metric facilitates regional and income-group comparisons across heterogeneous economic settings.

### Regional aggregation

Regional VLW and %GDP loss were calculated for five sociodemographic index (SDI) regions, seven GBD super-regions, and twenty-one GBD geographic regions by summing the corresponding national-level estimates. For each region r, we computed:

$$VLW_{r}=\$;\$\%GDP_{loss,r}=\left(\frac{\sum_{i\in r}VLW_{i}}{\sum_{i\in r}GDP_{i}}\right)x100$$

### Discounting and statistical implementation

A 3% annual discount rate^[25,26]^ to account for the time preference of future health losses, in line with standard health economic evaluation guidelines. Discounted DALYs were incorporated into a parallel analysis to estimate the present value of future lost life years and morbidity, allowing comparison with undiscounted estimates. Missing data were addressed using a comprehensive imputation approach primarily based on linear interpolation (for details, see Supplemental Digital Content Methods, available at: http://links.lww.com/JS9/G604). DALY estimates from the GBD 2021 database are reported with 95% uncertainty intervals (UIs)^[19,20]^, and corresponding uncertainty was propagated through the valuation of VSLY to capture the range of plausible estimates^[12,13,21]^.

All analyses were conducted using RStudio (RStudio PBC, Boston, MA, USA). This study adhered to the Consolidated Health Economic Evaluation Reporting Standards, ensuring methodological transparency and reproducibility^[27]^.

### Reporting guideline compliance

This manuscript adheres to the TITAN 2025 Guidelines (Transparency in the Reporting of Artificial INtelligence). No artificial intelligence technologies were employed at any stage of this study, including its design, data acquisition, statistical processing, interpretation of findings, or manuscript preparation and revision^[28]^.

## Results

### Global trends

In 2021, the median estimated VLW due to CRC globally was USD 3 493 688.30 million (95% UI: 3 022 928.60–3 964 775.59 million), accounting for approximately 2.28% (95% UI: 1.97%–2.58%) of the global GDP, reflecting the substantial impact of CRC on the global macroeconomic system (Table 1).

**Table 1:** VLW and VLW/GDP by GBD regions in 2021 for CRC, generated using income elasticity of the VSL at 1.00.

Region	VLW region (millions)	VLW region (millions)	VLW region (millions)	VLW/GDP(%)	VLW/GDP (%)	VLW/GDP (%)
		95% UI lower	95% UI upper		95% UI lower	95% UI upper
Global trends						
Global	3 493 688.30	3 022 928.60	3 964 775.59	2.28	1.97	2.58
Central Europe, Eastern Europe, and Central Asia	530 161.58	460 700.39	605 932.91	3.48	3.03	3.98
High-income	1 719 189.44	1 544 743.75	1 865 293.21	2.93	2.64	3.18
Latin America and Caribbean	193 212.67	169 821.11	218 163.38	1.64	1.44	1.85
North Africa and Middle East	68 454.96	53 959.74	86 165.95	0.75	0.59	0.94
South Asia	84 114.41	72 399.13	98 889.13	0.58	0.50	0.69
Southeast Asia, East Asia, and Oceania	867 447.41	696 437.26	1 051 112.81	2.20	1.76	2.66
Sub-Saharan Africa	31 107.84	24 867.22	39 218.20	0.64	0.51	0.81
5 SDI regions						
High SDI	1 702 375.66	1 527 121.89	1 855 375.67	2.81	2.52	3.07
High-middle SDI	1 360 257.07	1 140 151.21	1 593 257.52	2.68	2.25	3.14
Middle SDI	298 178.59	246 098.66	353 930.34	1.44	1.19	1.71
Low-middle SDI	122 773.81	102 239.19	148 613.60	0.62	0.52	0.76
Low SDI	10 103.18	7317.65	13 598.47	0.53	0.39	0.72
7 super-regions						
Central Europe, Eastern Europe, and Central Asia	530 161.58	460 700.39	605 932.91	3.48	3.03	3.98
High-income	1 719 189.44	1 544 743.75	1 865 293.21	2.93	2.64	3.18
Southeast Asia, East Asia, and Oceania	867 447.41	696 437.26	1 051 112.81	2.20	1.76	2.66
Latin America and Caribbean	193 212.67	169 821.11	218 163.38	1.64	1.44	1.85
North Africa and Middle East	68 454.96	53 959.74	86 165.95	0.75	0.59	0.94
Sub-Saharan Africa	31 107.84	24 867.22	39 218.20	0.64	0.51	0.81
South Asia	84 114.41	72 399.13	98 889.13	0.58	0.50	0.69
21 Geographic regions						
Western Europe	746 359.48	657 313.64	827 218.48	3.13	2.76	3.47
High-income North America	673 124.05	628 228.72	709 775.32	2.60	2.43	2.75
East Asia	671 220.65	540 292.71	812 117.34	2.31	1.86	2.79
Eastern Europe	326 341.77	286 695.07	369 266.14	4.00	3.51	4.52
High-income Asia Pacific	298 815.51	258 475.02	327 220.56	3.35	2.90	3.67
Central Europe	187 122.21	160 103.70	216 825.08	3.33	2.85	3.86
Southeast Asia	152 176.51	117 593.05	188 878.79	1.78	1.37	2.20
South Asia	84 114.41	72 399.13	98 889.13	0.58	0.50	0.69
Tropical Latin America	71 750.23	66 445.79	76 494.43	1.73	1.60	1.84
North Africa and Middle East	68 454.96	53 959.74	86 165.95	0.75	0.59	0.94
Southern Latin America	47 167.48	41 427.59	53 106.77	2.56	2.25	2.88
Australasia	43 609.81	38 207.67	49 567.32	2.49	2.19	2.83
Central Latin America	40 581.46	35 237.40	46 341.56	1.19	1.03	1.36
Andean Latin America	22 528.46	17 729.69	28 316.84	1.26	0.99	1.58
Central Asia	16 697.59	13 901.62	19 841.68	1.17	0.97	1.38
Southern Sub-Saharan Africa	11 798.09	10 383.36	13 585.07	1.37	1.20	1.58
Eastern Sub-Saharan Africa	8836.69	6855.30	11 695.36	0.71	0.55	0.94
Caribbean	7073.15	5548.04	8962.47	1.65	1.29	2.09
Western Sub-Saharan Africa	6987.92	5184.32	9147.45	0.34	0.25	0.44
Latin America and Caribbean	4111.89	3432.61	4941.31	3.04	2.54	3.65
Central Sub-Saharan Africa	3485.14	2444.23	4790.33	0.54	0.38	0.74
High-income	890.40	726.37	1078.85	5.01	4.08	6.07
Oceania	295.18	219.53	382.11	0.51	0.38	0.66
Southeast Asia, East Asia, and Oceania	145.25	124.30	167.25	1.85	1.58	2.13

CRC, colorectal cancer; GBD, Global Burden of Disease; GDP, gross domestic product; IE, income elasticity; SDI, sociodemographic index; UI, uncertainty interval; VLW, Value of Lost Welfare; VSL, Value of a Statistical Life.

### Global trends by SDI

**Figure 1.**
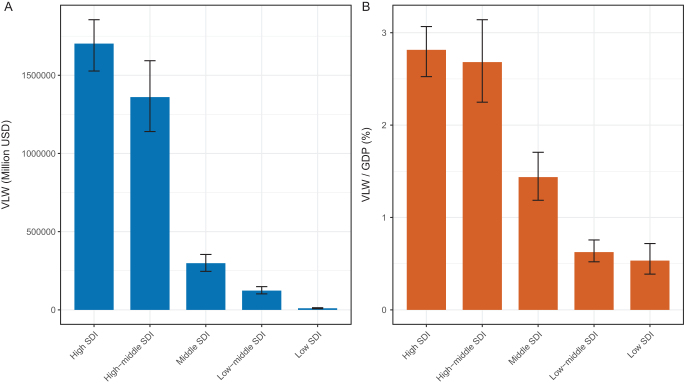
VLW and VLW/GDP by five SDI regions in 2021 for CRC, generated using IE of the VSL at 1.00 (A: VLW. B: VLW/GDP). CRC, colorectal cancer; GDP, gross domestic product; IE, income elasticity; SDI, sociodemographic index; VLW, Value of Lost Welfare; VSL, Value of a Statistical Life.

Notable disparity patterns were observed in CRC–related economic losses across five SDI regions. In low-SDI countries, the VLW was estimated at a median of USD 10 103.18 million (95% UI: 7317.65–13 598.47 million), accounting for 0.53% (95% UI: 0.39%–0.72%) of GDP. In low-middle-SDI regions, VLW reached USD 122 773.81 million (95% UI: 102 239.19–148 613.60 million), corresponding to 0.62% of GDP (95% UI: 0.52%–0.76%). Middle-SDI countries experienced a VLW of USD 298 178.59 million (95% UI: 246 098.66–353 930.34 million), representing 1.44% (95% UI: 1.19%–1.71%) of GDP. In high-middle-SDI regions, VLW was estimated at USD 1 360 257.07 million (95% UI: 1 140 151.21–1 593 257.52 million), equivalent to 2.68% (95% UI: 2.25%–3.14%) of GDP. High-SDI countries reported the highest losses, with a median VLW of USD 1 702 375.66 million (95% UI: 1 527 121.89–1 855 375.67 million), representing 2.81% (95% UI: 2.52%–3.07%) of GDP (Table 1, Fig. 1A, B).

### Global trends by super-region

In 2021, the highest economic losses due to CRC were observed in high-income regions, with a median estimated VLW of 1 719 189.44 million US dollars (95% UI: 1 544 743.75–1 865 293.21), representing 2.93% (95% UI: 2.64%–3.18%) of GDP. Southeast Asia, East Asia, and Oceania had the greatest relative burden, with a VLW median estimate of 867 447.41 million US dollars (95% UI: 696 437.26–1 051 112.81), accounting for 2.20% (95% UI: 1.76%–2.66%) of GDP. Central Europe, Eastern Europe, and Central Asia had a VLW of 530 161.58 million US dollars (95% UI: 460 700.39–605 932.91), representing 3.48% (95% UI: 3.03%–3.98%) of GDP. Latin America and the Caribbean suffered losses of 193 212.67 million US dollars (95% UI: 169 821.11–218 163.38), accounting for 1.64% (95% UI: 1.44%–1.85%) of GDP.

**Figure 2.**
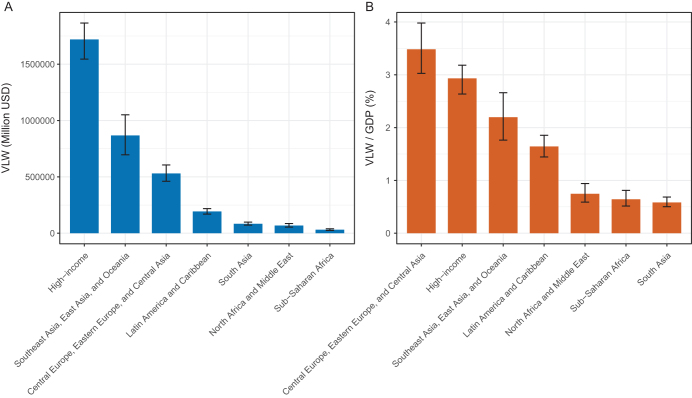
VLW and VLW/GDP by GBD super-regions in 2021 for CRC, generated using IE of the VSL at 1.00 (A: VLW. B: VLW/GDP). CRC, colorectal cancer; GBD, Global Burden of Disease; GDP, gross domestic product; IE, income elasticity; VLW, Value of Lost Welfare; VSL, Value of a Statistical Life.

In contrast, South Asia had a much lower absolute and relative economic burden, with a median VLW of USD 84 114.41 million (95% UI: 72 399.13–98 889.13), representing 0.58% of GDP (95% UI: 0.50%–0.69%). North Africa and the Middle East followed, with a VLW of USD 68 454.96 million (95% UI: 25 953.00–51 539.00) and a GDP share of 0.75% (95% UI: 0.59%–0.94%). The lowest burden was observed in Sub-Saharan Africa, where the VLW was only USD 31 107.84 million (95% UI: 24 867.22–39 218.20), accounting for 0.64% of GDP (95% UI: 0.51%–0.81%) (Table 1. Fig. 2A, B).

### Geographic regions

In 2021, the highest absolute economic losses due to CRC were observed in Western Europe, High-income North America, and East Asia. The median VLW was estimated at USD 746 359.48 million (95% UI: 657 313.64–827 218.48) in Western Europe and USD 673 124.05 million (95% UI: 628 228.72–709 775.32) in High-income North America, accounting for 3.13% (95% UI: 2.76%–3.47%) and 2.60% (95% UI: 2.43%–2.75%) of their respective GDPs. In East Asia, the VLW reached USD 671 220.65 million (95% UI: 540 292.71–812 117.34), corresponding to 2.31% of GDP (95% UI: 1.86%–2.79%).

In contrast, when assessing economic burden relative to regional GDP, the High-income Asia Pacific region showed the highest proportional burden, with CRC-related VLW accounting for 5.01% of GDP (95% UI: 4.08%–6.07%), despite a much lower absolute VLW of USD 89.04 million (95% UI: 72.64–107.89). Eastern Europe and High-income Asia Pacific followed, with GDP shares of 4.00% (95% UI: 3.51%–4.52%) and 3.35% (95% UI: 2.90%–3.67%), respectively.

**Figure 3.**
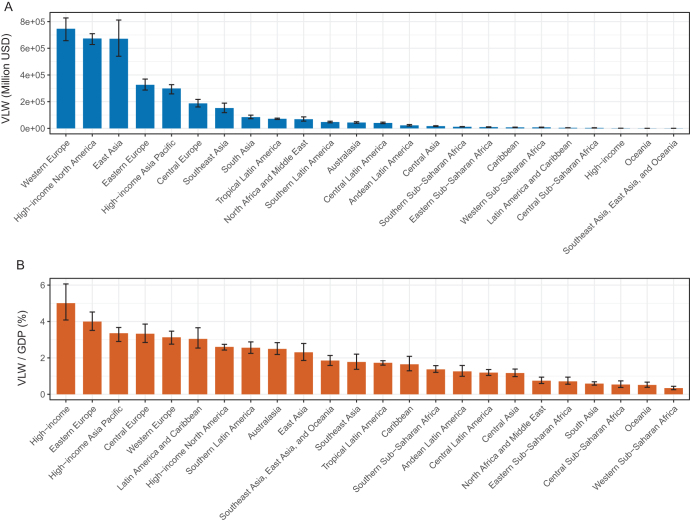
VLW and VLW/GDP by GBD geographic regions in 2021 for CRC, generated using IE of the VSL at 1.00 (A: VLW. B: VLW/GDP). CRC, colorectal cancer; GBD, Global Burden of Disease; GDP, gross domestic product; IE, income elasticity; VLW, Value of Lost Welfare; VSL, Value of a Statistical Life.

At the opposite end of the spectrum, Oceania exhibited a relatively low economic burden attributable to colorectal cancer, with a VLW estimated at USD 295.18 million, representing only 0.33% of the region’s GDP (Table 1; Fig. 3A, B).

### National trends

In 2021, under the non-discounted assumption, the estimated VLW due to CRC across 204 countries and territories ranged from less than USD 500 million (e.g., Tuvalu, the Republic of Nauru, and Kiribati) to over USD 0.6 trillion in countries, such as China and the United States. Across the 204 countries and territories, the median VLW was approximately USD 774.01 million [interquartile range (IQR): USD 144.99–7946.04].

The corresponding VLW as a proportion of national GDP varied even more substantially across countries, ranging from 0.19% (Republic of the Gambia) to 6.43% (Bulgaria), with a median of 1.37% (IQR: 0.66%–2.60%).

**Figure 4.**
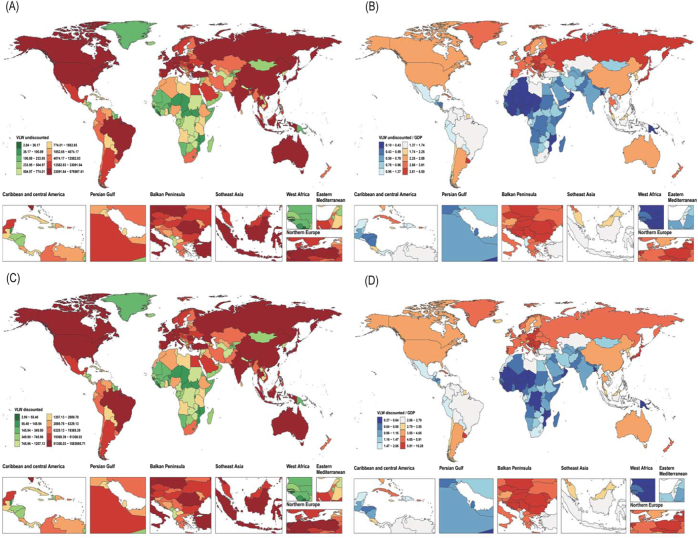
Estimated discounted and counted VLW and VLW as a percentage of GDP for colorectal cancer in 2021 across 204 countries and territories, based on the GBD study and assuming an IE of the VSL of 1.00. (A) Discounted VLW; (B) Discounted VLW/GDP; (C) Counted VLW; (D) Counted VLW/GDP. CRC, colorectal cancer; GBD, Global Burden of Disease; GDP, gross domestic product; IE, income elasticity; VLW, Value of Lost Welfare; VSL, Value of a Statistical Life

After applying a 3% annual discount rate, VLW estimates increased universally across countries, with an overall increase of approximately 35% to 67%. The global median VLW after discounting rose to USD 1207.13 million, and the median VLW/GDP ratio increased to 2.06%. Countries with the greatest increases due to discounting were primarily high-income economies with aging populations, including Singapore (66.54%), Japan (65.40%), and the Republic of San Marino (64.97%) (Supplemental Digital Content Table S1, available at: http://links.lww.com/JS9/G604; Fig. 4A–D).

### Sensitivity analysis

GBD region, country by-country estimates of VLW and VLW/GDP for CRC in 2021 using IEs of 0.55 and 1.5 are presented (Supplemental Digital Content Tables S2–S5, available at: http://links.lww.com/JS9/G604, and Supplemental Digital Content Figures S1–S6, available at: http://links.lww.com/JS9/G604), respectively. Globally, increasing the IE from 0.55 to 1.5 led to reductions in both VLW and VLW/GDP, consistent with the notion that higher elasticities down-weight valuations in lower-income settings. Across both IE scenarios, high-middle- and high-SDI regions exhibited the largest VLW/GDP ratios. At the super-regional level, under the IE = 1.5 scenario, the ranking of VLW/GDP across regions was broadly consistent with that observed under IE = 1.0. In contrast, under IE = 0.55, Southeast Asia, East Asia, and Oceania showed relatively higher VLW/GDP values. At the national level, China, the United States, and Japan consistently ranked among the top three in absolute VLW. After applying a 3% annual discount rate, high-income and aging economies demonstrated the most pronounced increases, reflecting a consistent discounting effect.

## Discussion

As global population aging accelerates and epidemiological risk factors persist, the burden of CRC is projected to escalate rapidly by 2040^[29,30]^. Given these challenges, quantifying the economic burden of CRC is essential to inform long-term health planning and resource allocation. Traditional studies have largely relied on the value of lost output (VLO) or DALY-based estimates, which mainly capture direct productivity loss or healthy life years lost, but often fail to reflect the broader societal welfare impact of disease^[31]^.

In contrast, this study applied the VLW approach, incorporating DALYs with the VSLY model to estimate both market and non-market economic loss^[31]^. By accounting for non-working populations (e.g., the elderly and unemployed), IE, and time discounting, this method offers a more comprehensive assessment of CRC’s macroeconomic impact, providing stronger evidence to guide priority-setting, fiscal planning, and equity-focused policy development.

### Global economic estimates and comparison

Consistent with previous epidemiological studies, our findings underscore the substantial burden of CRC across populations. In 2021, CRC was associated with a global economic loss exceeding USD 3.49 trillion, accounting for 2.28% of global GDP. This estimate is higher than most prior disease-specific economic evaluations^[12,32]^. For instance, a study published in JAMA Oncology estimated that CRC as the second-largest driver of economic burden among all cancers, with an estimated cumulative cost of USD 2.8 trillion over the 2020–2050 period^[12]^. Our findings for a single year highlight an even more immediate and acute economic pressure. This difference reflects methodological advances rather than overestimation. While traditional VLO or indirect cost methods focus on measurable market output losses (e.g., labor force decline)^[33]^, the VLW method captures a broader scope of welfare loss across all age and employment groups^[34]^.

Moreover, the VLW model incorporates IE (IE = 1.0) and time discounting, thereby broadening the scope of traditional economic evaluations. Sensitivity analyses using alternative IE values (IE = 0.5 and 1.5) yielded consistent results, supporting the robustness of the estimates. In addition, applying a 3% annual discount rate still produced substantial economic losses, with discounted VLW estimates increasing by 35%–67% and the global median VLW-to-GDP ratio reaching 2.06%, underscoring the cumulative and long-term welfare burden of CRC, particularly in aging populations.

### Regional disparities and national differentiation

To better understand the distribution of economic burden, we further analyzed CRC-related VLW-to-GDP ratios across regions and countries. The results revealed particularly high macroeconomic burdens in upper-middle- and high-income super-regions, with Central Europe, Eastern Europe, and Central Asia, and High-income areas ranking above the global average. This supports previous findings that middle- and high-income countries disproportionately shoulder the CRC burden^[3,35]^. While these countries often have well-established healthcare systems, their aging populations^[36]^ and persistent exposure to the adoption of western lifestyles^[37,38]^, and obesity^[39,40]^ continue to drive high CRC incidence and associated economic losses.

However, there are also differences within high-income countries. In 2021, the age-standardized incidence burden and economic burden of CRC in Monaco (discounted or undiscounted) were much higher than in other parts of developed countries, likely due to its small population size, highly aged demographic structure^[40,41]^. In contrast, some other high-income countries, such as the United Arab Emirates, have a less severe CRC burden due to a younger population structure (only 1.9% aged 65 and over in 2023)^[41]^. These inter-country differences suggest that even at similar income levels, differences in risk factor exposure and prevention and control measures can still lead to significant divergence in disease burden.

### Linking macroeconomic welfare losses to surgical capacity investment

Beyond prevention, our macroeconomic welfare loss estimates also suggest that inadequate surgical infrastructure and limited access to timely colorectal surgery may contribute substantially to the overall welfare loss. Surgery is a cornerstone of CRC management, yet approximately 5 billion people worldwide lack access to safe, affordable, and timely surgical and anesthesia care, with 80% residing in LMICs^[42]^. The Lancet Commission on Global Surgery estimated that the cumulative global economic losses from unmet surgical needs could reach USD 12.3 trillion between 2015 and 2030 if capacity scale-up does not occur^[43]^. Strategic investment in surgical systems could yield large economic returns, with modeling studies showing a potential ROI exceeding 14:1 in some LMICs^[44]^. Meanwhile, CRC screening remains underdeveloped in most LMICs, where organized programs are rare and participation rates remain below 10% compared with over 60% in high-income countries^[45]^.

Enhancing equitable access to early detection and timely surgery through the expansion of operating capacity, training of surgical teams, and strengthening of perioperative and pathology services may therefore reduce mortality, late-stage disease, and associated welfare losses^[46]^. These findings underscore that investing in surgical and screening capacity is both an economic and a health priority, capable of improving population outcomes, reducing disparities, and promoting sustainable growth in LMICs.

### Potential for preventive interventions

Given the substantial health and economic burden of CRC, identifying modifiable risk factors and promoting effective prevention strategies are essential. According to the GBD 2021 study, approximately 27.1% of CRC-related DALYs are attributable to behavioral or metabolic risk factors^[3]^, including alcohol use, smoking, physical inactivity, and high BMI. This suggests that primary prevention could substantially reduce CRC burden worldwide. Evidence also indicates that every US dollar invested in the prevention and control of noncommunicable diseases, including colorectal cancer, could yield a return of up to US$7 in health and economic benefits^[47]^, highlighting its exceptional cost-effectiveness. Priority should be given to improving dietary patterns, and strengthening early screening and intervention for metabolic syndrome and related conditions.

### Limitations and future directions

This study has several limitations. First, the estimates were based on GBD 2021 data, which rely on modeling and secondary inputs. In low- and middle-income countries with limited or poor-quality data, DALY estimates may carry uncertainty, potentially affecting the accuracy of VLW values. Second, although the VSLY method is comparable across countries, its key parameters (e.g., value of statistical life-year and IE) are mostly derived from high-income countries and do not fully account for cultural and institutional differences, potentially affecting its applicability and interpretability in specific regions. Third, this study primarily focused on welfare losses associated with mortality and did not include non-fatal or indirect impacts of colorectal cancer, such as long-term intestinal dysfunction, psychological distress, caregiving burden, and reduced productivity among survivors. This limitation partly reflects the structural constraints of the GBD framework, which quantifies health loss mainly through DALYs and lacks detailed information on indirect or intangible economic consequences. Therefore, the estimated VLW should be considered conservative. Future research should strengthen the collection of micro-cost data at the national level and integrate health economic models and behavioral policy studies. These VLW estimates can help policymakers and health economists identify priority interventions and allocate resources where they are likely to yield the greatest societal benefit, including investments in surgical infrastructure, colorectal cancer screening programs, and equitable access to treatment.

## Conclusion

This study, based on GBD 2021 data and a standardized VSLY approach, provides the first global assessment of the economic loss attributable to CRC. In 2021, CRC caused an estimated USD 3.49 trillion in losses, equivalent to 2.28% of global GDP. High and high-middle-SDI regions bore the greatest burden relative to GDP (2.81% and 2.68%, respectively), far exceeding that of lower-SDI regions (less than 1%), reflecting the compounding effects of aging and lifestyle transitions. These findings indicate that CRC represents a substantial health burden as well as a considerable economic challenge. Future strategies should integrate health equity and economic resilience by prioritizing cost-effective interventions and enhancing international cooperation and resource allocation.

## Data Availability

The datasets analyzed during the current study are available at https://vizhub.healthdata.org/gbd-results, and the World Bank’s World Development Indicators database (https://databank.worldbank.org/databases).
